# The Validation of a Web‐Based Self‐Administered Screening Tool for Dementia in Thai Older Adults

**DOI:** 10.1002/alz70860_103055

**Published:** 2025-12-23

**Authors:** Rapas Samalapa, Natthaphon Ubonsutvanich, Aisawan Petchlorlian, Tipanetr Ngamkala, Piangporn Charernwat, Pichayanan Wattanavitukul, Sirintorn Chansirikarnjana

**Affiliations:** ^1^ Geriatric Excellence Center, King Chulalongkorn Memorial Hospital, The Thai Red Cross Society, Bangkok, Thailand; ^2^ Faculty of Medicine, Chulalongkorn University, Bangkok, Thailand; ^3^ Division of Geriatric Medicine, Department of Medicine, Ramathibodi Hospital, Mahidol University, Bangkok, Thailand

## Abstract

**Background:**

The prevalence of dementia is increasing with the aging population. However, the rate of screening for cognitive decline is relatively low in Thailand due to a shortage of healthcare professionals and limited access to specialized services. We developed a voice‐based, **S**elf‐**A**dministered test for **M**emory, **O**rientation, and flue**N**cy for the **G**eriatric population (**SAMONG**), consisting of registration, orientation (1 point), fruit fluency (1 point), and 3‐word recall (3 points). The web‐based design of the test enhances accessibility, thereby boosting the screening rate. The aim of this study is to assess the diagnostic accuracy of SAMONG in identifying dementia in Thai older patients.

**Method:**

This cross‐sectional study enrolled patients aged 60 years and older, with and without dementia, classified by geriatricians according to the DSM‐5 criteria and the Clinical Dementia Rating (CDR). All patients used SAMONG in a standardized setting. The SAMONG results were interpreted automatically by the program algorithm, independently of the DSM‐5 criteria diagnosis. Diagnostic performance was analyzed using ROC and AUC. The accuracy of voice‐based automatic interpretation was compared with conventional human interpretation of recorded audio using Bland‐Altman analysis.

**Result:**

The diagnostic performance of SAMONG showed an overall AUC of 0.899 (0.863–0.934). The optimal cutoff score of ≥2 out of 5 yielded 97% sensitivity and 72% specificity. Fruit fluency alone demonstrated high performance in identifying dementia, with an AUC of 0.884 (0.836–0.932). A cutoff fruit score of ≥10 yielded 79% sensitivity and 85% specificity. Bland‐Altman analysis showed that the automatic interpretation of SAMONG had satisfactory agreement with human interpretation for both the total 4‐item test and individual items.

**Conclusion:**

SAMONG demonstrates excellent diagnostic accuracy in screening for dementia among Thai older adults. With its promising performance and potential for optimization through scoring adjustments, SAMONG has the capacity to enhance cognitive screening accessibility in broader populations and diverse settings. Its implementation could improve efficiency and reduce the burden on the healthcare system.

